# Magnetic Resonance Imaging Based Radiomic Models of Prostate Cancer: A Narrative Review

**DOI:** 10.3390/cancers13030552

**Published:** 2021-02-01

**Authors:** Ahmad Chaddad, Michael J. Kucharczyk, Abbas Cheddad, Sharon E. Clarke, Lama Hassan, Shuxue Ding, Saima Rathore, Mingli Zhang, Yousef Katib, Boris Bahoric, Gad Abikhzer, Stephan Probst, Tamim Niazi

**Affiliations:** 1 School of Artificial Intelligence, Guilin University of Electronic Technology, Guilin 541004, China; lama.hassan@etu.unilim.fr (L.H.); sding@guet.edu.cn (S.D.); 2 Lady Davis Institute for Medical Research, McGill University, Montreal, QC H3S 1Y9, Canada; bbahoric@jgh.mcgill.ca (B.B.); gad.abikhzer@mcgill.ca (G.A.); sprobst@jgh.mcgill.ca (S.P.); 3 Nova Scotia Cancer Centre, Dalhousie University, Halifax, NS B3H 1V7, Canada; Mike.Kucharczyk@nshealth.ca; 4 Department of Computer Science, Blekinge Institute of Technology, SE-37179 Karlskrona, Sweden; abbas.cheddad@bth.se; 5 Department of Radiology, Dalhousie University, Halifax, NS B3H 1V7, Canada; SharonE.Clarke@nshealth.ca; 6 Center for Biomedical Image Computing and Analytics, University of Pennsylvania, Philadelphia, PA 19104, USA; saima.rathore@pennmedicine.upenn.edu; 7 Montreal Neurological Institute, McGill University, Montreal, QC H3A 2B4, Canada; mingli.zhang@mcgill.ca; 8 Department of Radiology, Taibah University, Al-Madinah 42353, Saudi Arabia; ykatib@taibahu.edu.sa

**Keywords:** artificial intelligence, radiomics, radiogenomics, prostate cancer, Gleason score, magnetic resonance imaging

## Abstract

**Simple Summary:**

The increasing interest in implementing artificial intelligence in radiomic models has occurred alongside advancement in the tools used for computer-aided diagnosis. Such tools typically apply both statistical and machine learning methodologies to assess the various modalities used in medical image analysis. Specific to prostate cancer, the radiomics pipeline has multiple facets that are amenable to improvement. This review discusses the steps of a magnetic resonance imaging based radiomics pipeline. Present successes, existing opportunities for refinement, and the most pertinent pending steps leading to clinical validation are highlighted.

**Abstract:**

The management of prostate cancer (PCa) is dependent on biomarkers of biological aggression. This includes an invasive biopsy to facilitate a histopathological assessment of the tumor’s grade. This review explores the technical processes of applying magnetic resonance imaging based radiomic models to the evaluation of PCa. By exploring how a deep radiomics approach further optimizes the prediction of a PCa’s grade group, it will be clear how this integration of artificial intelligence mitigates existing major technological challenges faced by a traditional radiomic model: image acquisition, small data sets, image processing, labeling/segmentation, informative features, predicting molecular features and incorporating predictive models. Other potential impacts of artificial intelligence on the personalized treatment of PCa will also be discussed. The role of deep radiomics analysis-a deep texture analysis, which extracts features from convolutional neural networks layers, will be highlighted. Existing clinical work and upcoming clinical trials will be reviewed, directing investigators to pertinent future directions in the field. For future progress to result in clinical translation, the field will likely require multi-institutional collaboration in producing prospectively populated and expertly labeled imaging libraries.

## 1. Introduction

Prostate cancer (PCa) is the most common non-skin cancer in men, presenting a global healthcare challenge [1,2]. Management strategies range from active surveillance, a definitive surgical intervention, or a radiotherapy approach, which may entail years of antiandrogen therapy. Selecting how to manage these patients is heavily dependent on the PCa grade, a biomarker for its underlying biological aggressiveness. A patient with low risk PCa is likely to do well regardless of the management strategy employed [3]. In contrast, high risk PCa carries a significant likelihood of treatment failure even if a more intense and prolonged therapy is undertaken [4].

Presently, PCa is diagnosed and its grade is evaluated via invasive biopsy. The biopsied specimen is assessed by a pathologist to establish the grade. The grade itself is most commonly reported as the Gleason score (GS), a sum of two ordinal classifiers of the most predominant grades visualized by the pathologist, typically ranging from 6 to 10 [5]. More recently, GS values have been standardized by the International Society of Urological Pathology (ISUP) into an ordinal classifier ranging from 1 to 5 instead—the Grade Group [6]. As both are reported in the radiomics literature, it is worthwhile to note that while lower values predict for lesser lethality, similar values are not necessarily exchangeable between the two scales (i.e., Table 1).

However, prostate biopsies have multiple known limitations. Biopsy is frequently not reflective of the true grade [7], which may be due to sampling error [8], interobserver variability [9], and/or expertise [10]. Reported biopsy risks include pain, bleeding, erectile dysfunction, and infection [11,12,13,14]. Finally, biopsy also incurs costs secondary to assessments by multiple specialists and the patient’s other indirect expenses.

Imaging technologies partially address issues with sampling error. Combining magnetic resonance imaging (MRI) with ultrasonography (US)-guided biopsies [15,16,17] can facilitate sampling of the most suspicious regions. Multiparametric MRI (mpMRI) has advanced this approach; an MRI-targeted biopsy is less likely to miss more advanced PCa [18,19,20] and decreases the frequency of repeat biopsies [21]. Clinically, the European Association of Urology strongly suggests that imaging modalities, such as mpMRI, be considered prior to proceeding to biopsy when the pretest probability of prostate cancer being present is low [22].

Radiomic models offer a non-invasive reproducible method to assess PCa aggressiveness. Imaging characteristics, called textures or features, extracted from the labeled region of mpMRI can be utilized as an input for conventional classifier models [23,24]. Such radiomic models must select the most informative features using feature selection technique(s), otherwise the results may be biased by overfitting [25]. While this strategy has been well demonstrated in multiple malignancies [23,26,27,28], the underlying understanding of the most informative features and predictive models remains limited [29].

The growing interest in AI techniques and their applications in medicine [30], has carried over to computer-aided diagnostic (CAD) systems to detect, grade, and introduce other classifications of PCa [31,32,33,34,35,36]. So far, the term of radiomic with AI represents the features extraction and interpretation of hidden quantitative imaging data to be used for CAD [37]. To date, there has been a focus on conducting proof of concept studies. Radiomic models have been used to discriminate low from higher-grade PCa [38,39], directly predict the GS [23,24,40,41], lesion identification [42,43], and plan radiotherapy [44,45,46]. More recently, radiomic models have been utilized to predict genetic characteristics, a field known as radiogenomics. These studies have explored the potential in characterizing a PCa’s underlying biological aggression [47,48,49,50,51,52].

This narrative review synthesizes the current standards and state-of-the-art applications of radiomics for the classification of PCa. This includes our identification of the radiomic features with the greatest present significance and a description of the relation between metrics, techniques, and MRI sequences.

## 2. Multiparametric MRI (mpMRI) of Prostate Cancer

mpMRI is a type of non-invasive imaging integrating traditional anatomical sequences-triplanar T2-weighted images (T2W) and perfusion imaging, namely the diffusion-weighted images (DWI) with apparent diffusion coefficient maps (ADC) and T1-weighted imaging (T1W) for the generation of dynamic contrast-enhanced images (DCE) [53]. Alternative MRI sequences have also been evaluated for the PCa imaging, such as proton magnetic resonance spectroscopic imaging (MRSI) [54]. Owing to the greater acquisition time and extensive post-processing data required by MRSI, the DWI and DCE series are a preferred method to evaluate patients suspected of having PCa or stage those with biopsy-proven disease [55].

There is not a uniform consensus that mpMRI is required. Expertly interpreted biparametric MRI, which forgoes inclusion of DCE images, has been observed to be adequate to detect clinically significant PCa in a prospective cohort study [56]. A retrospective cohort study has suggested that the advantage of adding DCE may be of the greatest yield in the peripheral zone, the most common region for PCa to develop [57]. Regarding the radiomics pipeline, mpMRI offers a potential advantage at the level of feature extraction as well (see Section 3.5). With additional images to extract data from, there would be an increased likelihood of extracting a radiomic feature of significant predictive value.

Human interpretation of mpMRI, when incorporating a combined interpretation of T2W, DWI, and/or DCE series, can facilitate PCa detection. Clinically, mpMRI is used for tumor detection, active surveillance, and to aid in management decisions [53,58]. Though retrospective work may suggest a high specificity and sensitivity [59], a meta-analysis has been performed in populations with a higher pretest probability of having PCa. Pooled estimates observed that the sensitivity may be comparably high (82–96%) though specificity is likely far lower (33–71%) [60,61]. Positive predictive values of 98% have been obtained in limited retrospective series, but these high levels of fidelity only allowed for relatively rudimentary classifications (i.e., PCa versus benign) [62]. A more thorough investigation via meta-analysis observed that the positive predictive values ranged significantly between studies, ranging from 35 to 50% [60,63]. Appreciating the moderate clinical confidence imparted by these metrics, there would be an understandable need for technology that could allow for a reliable non-invasive prediction of the presence of malignancy and its grade. Important to note is the limits to generalizing these existing studies, as they speak to the evaluation of specific nodules rather than the whole prostate.

Heterogeneous mpMRI image composition presents further difficulties, largely due to a substantially diverse implementation of equipment across institutions [64]. To facilitate a standardized assessment of PCa, the European Society of Urogenital Radiology (ESUR) developed Prostate Imaging Reporting and Data System (PI-RADS) in 2012 [65,66,67], which was updated in 2015 (i.e., PI-RADS v2 [66]) and more recently in 2019 (i.e., PI-RADS v2.1 [68]). The output of this evaluation is an ordinal risk score between 1 and 5. Though PI-RADS allows for acceptable interobserver variability at expert centers [69], it does not address the issue in community settings [70]. Importantly, while it may allow for some reliable distinction between low- and high-grade malignancies [60,61,71], there has not been a demonstration that human interpretation reliably ascertains the GS. PI-RADS also does not overcome issues regarding the multifocality nor temporal and spatial intratumoral heterogeneity of PCa [23,72,73,74]. While PI-RADS sets multiple imaging standards, greater standardization of additional image acquisition details is necessary if the field is advancing to implement imaging characteristics not discernible by human evaluation. This requires a common acquisition protocol to standardize the image and avoid the heterogeneity in imaging quality.

Furthermore, other factors could alter mpMRI image acquisition on a daily or patient-to-patient basis, such as distortion related to the local magnetic field inhomogeneities due to rectal air or metal implants [75]. Diagnosis based on mpMRI suffers from interobserver variability, influenced by experience [76], and subtleties in differentiating benign and premalignant lesions that may closely resemble PCa [77]. Studies of AI-based radiomics have suggested that these models may become a reliable and informative biomarker complementary to human interpretation of mpMRI [23,24,31].

## 3. Radiomics Pipeline for Predicting Tumor Grade

### 3.1. Basic Flowchart

**Figure 1:**
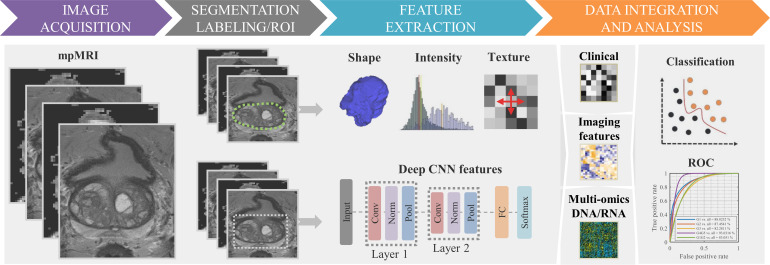
Flowchart of the standard radiomics model. (1) Multiparametric MRI (mpMRI) image acquisition. (2) Segmentation: tumor labeling-green/white contour. (3) Imaging features extraction using shape, texture, and/or deep features derived from convolution neural network layers. (4) Clinical, radiomic features, molecular data for statistical analyses, based significance test and classifier models, to identify relevant features for predicting the clinical outcome (e.g., Gleason score).

Several studies have utilized a standard pipeline for radiomic analysis, including the following main steps: image acquisition, segmentation (or labeling), feature extraction, feature selection, and statistical and predictive modeling [41,78,79,80,81]. Figure 1 illustrates the process of radiomic analysis as it pertains to identifying signatures for establishing the PCa grade group, as previously implemented by Chaddad et al. [23,24]. The product is a radiomic signature (a vector), which includes the most predictive features as its elements. This section outlines the application of radiomics for predicting a specific biomarker, the grade group, though a similar pipeline could be applied to predict a different clinical or molecular biomarker.

First, a database of a large number (e.g., preferred to be greater than 1000) of medical images (mpMRI) is prepared so that a set of standardized images can be subject to radiomic analysis with minimal bias [82,83,84]. The number of imaging features number is preferred to be equal or less than the number of samples. Prospective works validating a specific threshold of MRI images are lacking, though related work with computed tomography imaging has supported that thousands of medical images would likely be required [85]. Second, segmentation of images identifies regions of the image thought to be PCa as regions of interest (ROIs). Segmentation may be accomplished, manually or semi/fully automatically. Third, feature extraction records imaging features (e.g., standard features: shape descriptors, histogram statistics, texture; deep features, etc.) in one or more separate vectors for subsequent analysis. Fourth, radiomic features have their predictive capacity estimated (e.g., what is the relative importance of different radiomic features). Finally, univariate analysis (e.g., significance test, Spearman correlation, etc.) and multivariate analysis (e.g., models of classification and regression: random forest and logistic regression models) characterize models that exploit the earlier imaging features to predict the PCa. This final step should be done in a validation cohort of patients to demonstrate some measure of generalizability of the newly generated radiomics model.

In addition to radiomic features, clinical and molecular variables can readily be included in the eventual prediction model. Such details are thought to benefit predictions of the GS [86] but are also included in the radiogenomic studies. In these cases, imaging features are modeled to predict molecular characteristics (e.g., androgen resistance) or are combined with multiple biological features (e.g., genomics, proteomics, and metabolomics) to better predict a PCa’s potential aggressiveness.

Given the multidisciplinary expertise required to validate the different aspects of the radiomics pipeline, collaboration is essential. For example, oncologists will have input as to the clinical parameters to model and format of the output, radiologists can provide expert segmentation of the ROIs, molecular scientists may contribute genomic or proteomic variables, biomedical scientists can translate the clinical dilemma into a scientific question addressable by a machine-learning based approach, and those with statistical expertise can appropriately model the variables for the desired outcome. The interaction between disciplines is numerous, necessitating clear communication so that the eventual output has the potential to resolve the actual clinical question.

### 3.2. Image Acquisition

MRI radiomics have demonstrated the potential to discern the PCa grade [23,24,86,87,88] or guide management approaches [45,89] from the abundance of clinical data acquired at each scan. However, reproducibility is a significant issue at different stages of the radiomics pipeline, with few studies investigating this question [41,78]. At present, it is unknown if a radiomic model can be generalized to other patients imaged with the same scanner. While some imaging features are felt to remain stable between image acquisition events, more elegant solutions, such as image normalization, have failed to address the issue.

To investigate this problem, attention must be paid to the reported processing configuration in radiomics studies (or an emphasis must be placed on its reporting by potential peer reviewers and editors). Standardization of MRI image acquisition across vendors (e.g., Siemens, GE, Philips, Hitachi, etc.) offers an ambitious solution to reduce this variability, but understandable conflicts in revealing corporate intellectual property may limit complete transparency. Machine inherited artifacts and the discrepancy between scanners’ measurements are also acknowledged in the field of breast cancer (e.g., utilizing X-ray scanners) [90]. Pending studies must address the issue of radiomic feature stability, investigating if some features can remain stable between imaging events, so that collaboration and eventual widespread clinical implementation can be fostered.

### 3.3. Image Quality Assessment and Standardization

When images are acquired using multiple MRI scanners with various acquisition parameters (e.g., echo time, repetition time, flip angle, etc.), image quality can be very different. To first ensure that the acquired images are of sufficient quality, numerous methods have been proposed [91]. The most popular is intensity normalization, which uses a histogram of MRI images based on the background intensity only without the requirement of prior knowledge to assess image quality [92].

To develop a radiomics model that appropriately compares its acquired data, inputs must be standardized. Typical variations between MRI scanner models could be revealed in the following image parameters: pixel size, slice spacing, image contrast, slice thickness, patient location or variations introduced by reconstruction algorithms. By resampling to a standard resolution, typically 1 mm^3^ voxel resolution and an image size of 256 × 256 × slices (or 512 × 512 × slices, i.e., slices represent the third image dimensions) voxels, many of the aforementioned parameters will be standardized. Following this, signal intensities within each image are linearly transformed (normalized) to either the [0, 1] or [0, 255] range. There are also many other approaches to normalization-Gaussian and Z-score normalization are two common alternatives [93,94]. The normalization process will impact the values of the different radiomic features, influencing the information represented by each image and potentially interobserver reliability [95]. As multiple groups strive to optimize this process, an approach proposed in other disease sites [96] and AI-implementing clinical studies [97] forms a collaborative group to standardize a methodology to allow for ongoing intergroup comparison and collaboration.

### 3.4. PCa Segmentation

To investigate PCa imaging features via a standard radiomic analysis, an ROI corresponding to the tumor volume-region must first be segmented. Manual (or semiautomatic) segmentation is usually performed by specialized clinicians (i.e., diagnostic radiologists) [98]. The process of manual segmentation is subject to inter-rater variability, due to heterogeneity in the segmentation methodology employed between clinicians [99] and due to occasional physical fatigue. A common strategy used to overcome this inter-rater variability issue is incorporating the overlapping/common ROI of 2–3 segmentations, also called masks or labels, as the ground truth.

Many tools are available for segmentation, such as the publicly accessible 3D Slicer [100] or ITK-SNAP [101]. Once the ROI has been defined across all of the mpMRI images, there must then be a coregistration step that matches the tumor mask to the remaining mpMRI sequences (e.g., T2W, ADC, DCE, etc.), often by using the same segmentation tool [102,103]. This coregistration process is performed slice by slice on a single MRI sequence, known as the reference image. Most frequently, this is an axial T2W sequence. Any bias introduced due to an error in registration (alignment) is referred to as an image distortion inherent to DWI and the use of different image spatial resolution. Alternatively, the coregistration step has been foregone by segmenting each MRI-sequence individually, minimizing the potential for distortion [23]. Investigations comparing the consequences of distortion on the ultimate clinical classifier hold merit, validating the need for ROI localization with the highest fidelity.

Another segmentation strategy is fully automatic segmentation. The relative success of automatic segmentation is typically expressed as a Dice score or Dice similarity coefficient (DSC), quantifying the degree of overlap between the predicted mask and the ground truth [104]. DSC values range from 0 to 1, with a DSC of 1.0 communicating that there is a perfect overlap of the predicted segmentation and the truth, the ideal score. Values decrease as there is more discordance between the two, with a DSC of zero communicating there is no overlap. This approach has been demonstrated using classifier models with the prostate labeled on mpMRI images (i.e., T1W and T2W). For instance, the unsupervised learning utilized fuzzy c-means clustering was used for partitioning data into groups to achieve an average DSC of 0.91, relative to manual segmentation [105].

Advanced deep learning algorithms have deployed convolutional neural network (CNN) to segment the ROI corresponding to a PCa [106,107,108,109,110,111,112,113,114]. The most common model used is the U-Net architecture, which is proposed for fully automatic segmentation of PCa with a DSC of ≥0.89.

Without knowing where the limitations in segmentation exist, as a machine-learning based process does not necessarily have a predictable pattern in its “error”, it awaits further segmentation studies to determine if such DSCs are clinically adequate. If the continued refinement of CNNs has these values approach 1.00, the chance of any residual difference being clinically meaningful is low. To validate such an assumption, clinical studies will be essential.

### 3.5. Image Feature Extraction

Extracting image features from the ROI is arguably the principal step in radiomic analysis. Image features summarize the image information by elements vector to then be analyzed and/or be used as inputs for classifier models. Specifically, the imaging features encode the characteristics of the ROIs to describe their heterogeneity. Most types of imaging features will be based on their texture (e.g., gray-level co-occurrence matrix (GLCM), neighborhood gray-tone difference matrix (NGTDM), neighboring gray-level dependence matrix (NGLDM), gray-level run-length matrix (GLRLM), etc.) [115,116], shape (known as morphological features) [38], histogram-based descriptors [116], or features derived from deep CNN [117].

Among other novel imaging features based on texture computation, the joint intensity matrix (JIM) has been suggestive of greater predictive capacity for the GS. JIM derived features encode the spatial relationships of pairs of voxels derived from the corresponding pair of MRI sequences [23]. This approach outperforms models based on standard GLCM-derived features alone, which are only extracted from a single MRI sequence [24]. Showing great potential is a recent study describing how deep CNNs can generate deep texture features in PCa [117] or benign disease cases [118]. The ability to generate a multitude of features increases the likelihood of discovering imaging characteristics representative of the GS. This pipeline model was expanded by Chaddad et al., adapting multiple 2D CNN models to generate deep texture features in prostatic mpMRIs, generating a robust model for predicting the GS [88].

### 3.6. Feature Analysis and Prediction Model Construction

The features extracted from each image are aggregated as a vector, which is then subjected to further analysis. Either all or a preselected features are evaluated for their potential to be a non-invasive marker (alternatively, an indicator) associated with a clinical variable (e.g., molecular markers [50], GS [23], survival [119], and risk of breast cancer [90,120]). The term, radiomics, is representative of the various associations between an imaging feature and the clinical variable of interest. Similarly, radiogenomics specifically investigates the potential associations between imaging features and characteristics typically attributed to the genomics domain and its immediate derivatives (e.g., genotypes, gene expression profiles, and protein expression).

Aggregated features are then screened for candidates with the greatest likelihood to have a meaningful association with the clinical variable of interest. Typically accomplished via univariate analysis, imaging features are normally first assessed for rudimentary associations; namely, do they differ when the clinical variable changes (e.g., *T*-test and Wilcoxon test) or does the extent of that difference have a linear association with variations in the clinical variable (e.g., the Spearman correlation rank between the ROI’s entropy and the PCa’s GS). Once adjusted for the confidence in these estimates to correct for multiple sampling, often via the relatively strict Holm–Bonferroni correction [121], there will often be a limited number of candidate radiomic features remaining. The remaining features with the greatest and sufficient predictive capacity will be later in a multivariate model, being modeled with other radiomic features or clinical variables. Though the specifics of the predictive modeling are immensely diverse, the process of imaging feature extraction, evaluation, and implementation is representative of a standard radiomic model.

Predictive models [122,123] can incorporate covariates from a variety of sources (e.g., clinical, molecular, and radiomic [124]) to predict a clinical outcome. Deep learning models (e.g., CNNs) form a specific approach that is directly applied on images to extract, select features, and predict the class (classification) or a value (regression) in an automated fashion. Examples in the PCa literature have observed that this deep learning approach detects malignant lesions [125], predicts the GS [126], and segments the ROI [127,128].

A key limitation of deep learning approaches is the vast number of sample images required to robustly train a model (i.e., thousands of labeled data sets), presenting an often-insurmountable barrier to clinical translation. An approach to circumvent this limitation has been proposed to construct CNNs pretrained in other settings and then apply them to the clinical setting of interest [118,129,130,131,132]. In PCa specifically, Chaddad et al. used this approach to predict the GS with robust outcomes albeit with a smaller publicly available data set [88]. The established CNNs were trained on brain MRI data and used to generate multiscale texture of PCa images. Shannon entropy function is then used to encode the CNN features and transform them to a set of informative features called deep entropy features (DEFs) that were used as inputs to random forest classifiers to predict the GS of PCA.

Table 2 reports on the recently published works utilizing mpMRI to predict the GS. The inclusion of more classifying options by Jesen et al. [133] and Chaddad et al. [88] may be associated with the seemingly greater area under the ROC curve (AUC) values, implying some value to this approach. Common to many studies, frequent radiomic features used in GS predictions were based on texture (e.g., histogram, GLCM, NGTDM, and GLSZM), shape/morphological (e.g., volume and surface), and clinical markers (e.g., age and treatment modality). This is consistent with a recent survey that reports a median AUC value of 79% (IQR—interquartile range: 0.77–0.87) for PCa classifications [87].

However, metrics based on the true negative rate (i.e., background voxels correctly classified as cancer-negative) are affected by problems of class imbalance, which may occur if there is a large imbalance in the number of voxels within each class [134,135]. The aforementioned implementation of ROC curves and accuracy, commonly employed in the biomedical literature, suffer from such bias. To circumvent this bias, alternatives include precision–recall curves and DSCs instead [136].

## 4. Prostate Cancer Radiogenomics

Radiogenomics integrates imaging features to predict specific genomic characteristics, offering the potential to direct PCa therapy selection (i.e., a predictive biomarker) and guide informed decision-making (i.e., a prognostic biomarker [141]. There is a significant clinical impetus for advancing the field of radiomics in this direction. For example, the presence of specific double-strand DNA break deficiencies (i.e., BRCA 1 or 2) can predict the benefit of certain classes of drugs in metastatic castrate-resistant PCa [142]. Conversely, ad hoc analyses of a smaller phase II trial has suggested that specific genetic markers for a PCa’s genomic integrity (e.g., ATM, BRCA1/2, RB1, and TP53) could predict when aggressive local treatment of a metastatic disease may not enable a safe deferral of initiating symptomatic lifelong endocrine therapy [143]. Among ongoing randomized trials, prospective investigations of radiogenomic signatures could potentially allow for the identification of early androgen resistance, selecting populations that may (or may not [144]) benefit from intensification with novel androgen receptor axis therapies [145].

There has been a vast multitude of genetic characteristics identified as potentially relevant biomarkers in phylogenetic analyses [146,147] and our maturing understanding of epigenetics [148]. There is also significant discussion surrounding how additional biomarkers of a PCa’s molecular function could assist in determining how aggressive to be in the setting of early metastatic disease. There would be great value in delineating where an advanced PCa is in its natural history [149]. Different uncertainties and management strategies exist for potentially curable high-risk PCa versus oligometastatic hormone-sensitive disease, where long-term outcomes are being actively explored, versus an advanced castrate-resistant malignancy with a more rapid and inexorable course.

Radiogenomics has been studied more extensively in other malignancies, including central nervous system oncology (e.g., identifying high grade [28,150] and lower-grade gliomas [151]), lung cancer [152], and other tumors [153,154]. In PCa, investigations of a single gene have best characterized phosphatase and tensin homolog (PTEN) expression, a tumor suppressor of the AKT/PKB pathway [155]. Examples have included imaging features and the GS of a peripheral zone PCa having a weak but significant-association with PTEN expression [156] or that low ADC values correlated with PTEN expression, while PTEN expression was negatively correlated with the presence of lymph node involvement [157].

With advances in genome sequencing, there have been efforts instead to model the genomic profile of a PCa with the assistance of radiomic features, rather than studies characterizing just a limited genetic profile. Earlier studies utilized mpMRI alone in a small number of patients—six participants in two notable cases. The first related 49 conventional radiomic features to the GS and 65 genes evaluated among commercially available prostate cancer genomic assessments [124]. This exploratory study observed multiple radiomic features had significant correlations with gene expression. The second study profiled both abnormal and normal regions of their prostates with whole-exome DNA sequencing, identifying the mutational burden of cancer-associated genes profiled by the geographic region. Their radiogenomic modeling could separate GS 3 + 4 from GS 4 + 5 cancers, distinguishing potentially intermediate-risk from high-risk disease, but not predict the mutational load by region.

Radiogenomic signatures may be able to predict comprehensive PCa gene expression profiles from biopsy samples, rather than just their single gene constituents [158]. Since such gene expression has recently been suggested to not be as vulnerable to sampling bias/tumor heterogeneity as previously thought [159] and that PIRADS classification can predict for gene expression [160], the signal that prostate radiogenomics could potentially predict and identify validated genechip results is becoming more plausible. Retrospective works have suggested that dichotomous classifications (i.e., high or low scores) of the decipher genetic risk profile could be predicted from mpMRI with modest confidence (AUC = 0.80–0.84) and may be more reliable than predictions of the GS [161,162]. Other relevant explorations with positive signal have been of radiomic signatures, which distinguish the genomic profiles associated with high-risk pathological variants (e.g., intraductal carcinoma), [163] or hypoxic lesions of PCa [164].

Additional clinical sources of data have enriched radiogenomic analyses by combining novel functional imaging (prostate specific membrane antigen positron emission tomography/computer tomography (PSMA PET/CT)) or more classical clinical features with mpMRI [165]. Among five patients, there were multiple radiomic signatures in the index lesions that correlated with the number of copy number alterations, a measure of the PCa’s mutational load and underlying biological aggression [166]. Such a clear correlation was not observed by the earlier studies, which utilized mpMRI alone, though a contrast to the copy number alterations in normal tissue was not provided. The utility of PSMA PET/CT has also been supported by its ability to detect PTEN-loss with hopeful sensitivity (0.80) and specificity (0.77) among prostatectomy patients [167]. In a separate retrospective study of 298 prostate cancer patients that had undergone prostatectomy, they utilized earlier established conventional radiomic features [124] to predict the tumor grade. Unlike the other studies referenced in this selection, this was not an example of a radiomics model predicting genetic expression. They observed that by combining known genomic, radiomic, and clinical features, they improved the accuracy of their predictive model for the definitive tumor grade [47], also demonstrating possible evolution in the technical definition of the term radiogenomics.

An interesting clinical feature, which has spurred subsequent questions in radiogenomic analyses, is the visibility of a lesion on mpMRI. It has been appreciated that a greater proportion of lower grade PCa may be occult on mpMRI, relative to higher risk disease [168,169]. Multiple retrospective radiogenomic studies have supported that visible mpMRI lesions, and the genes that predict for visibility, do indeed represent a greater risk to the patient [163,170,171]. However, it is crucial to explore the nature of these infrequent but higher-risk occult mpMRI—what if the highest risk lesions would not even be segmented in the radiomics pipeline because they are not detectable?

Subsequent characterizations of the genomic profiles of mpMRI visible or invisible lesions have revealed biases in our potential other radiomic studies, as the invisible lesions will not have been included. For example, a retrospective study of 62 PCa lesions noted that among 5 evaluated genes, all CHD1 overexpressing lesions were invisible to mpMRI [172]. A comprehensive retrospective study of intermediate-to-high risk PCa patients utilized genomic, epigenomic, and transcriptomic data to appreciate trends among 43 prostate core samples from 6 different patients compared to both malignant and benign nodules [173]. Though limited in number, three out of six cores in mpMRI invisible lesions contained at least one mutation thought to be representative of a more advanced disease state from robust phylogenetic studies (e.g., multiple DNA repair genes) [146].

Though it would not address the issue of bias introduced from earlier studies, there is reassurance offered from a retrospective study, which produced a radiogenomic signature for tumor visibility among 10 patients with 26 PCa lesions [174]. The resultant nine-gene signature generated was modestly sensitive and very specific, 75 and 100% respectively (AUC = 0.88) for mpMRI visible lesions and did not seem to be prognostic for poor outcomes. This radiogenomics study did however emphasize the need to develop a method to evaluate invisible mpMRI lesions.

As this review continues to suggest directions for scientific exploration, attention should be drawn to present gaps in the radiogenomic literature. To date, there is no published prospective work on the utility or validity of radiogenomics. Next, the number of potentially clinically relevant biomarkers is likely to expand as various other -omic profiles (e.g., Metabolomics and proteomics) gather the evidence necessary to felt to complement our assessment of PCa. To date, the radiomic features used in these analyses have not evaluated the yield of deep radiomic features. Finally, models that consist of biomarkers of multiple sources (e.g., clinical, radiomic, and genomic) have repeatedly demonstrated improved predictive capacity. Appreciating these relative deficits can help the field appreciate where the most pertinent radiogenomic inquiries can be pursued.

## 5. Barriers and Strategies for Clinical Translation

Outside of technical refinement and a greater volume of imaging data, there remain significant issues of bias and generalizability that must be overcome before predictive radiomic models can be clinically implemented in the initial assessment of PCa. The issue of generalizability pertains to both heterogeneity in image acquisition parameters and uncertainty surrounding the stability of imaging features [78]. A prospective study, conducted to respect consortium guidelines [66,97], will be required to address the generalizability issue. It must demonstrate that the radiomic model is sufficiently resilient to instability in its radiomic features following serial assessments. Given the present state-of-the-art, such a study is unlikely to suggest that radiomic models are ready for clinical implementation to be positive. To advance the radiomics field, the study would need to discover novel radiomic features that remain stable between serial assessments—a feasible outcome given the success of CNNs in this role [88]. If sufficient stable features were identified, this would lead organically to a multicenter validation study that could demonstrate intra- and interpatient radiomic feature stability.

Complicating matters is the quality of the gold standard. The majority of PCa radiomic studies treat prostate biopsy as the gold standard evaluation of the GS, as per the clinical guidelines, which utilize biopsy results to determine a management strategy [175]. Unfortunately, in approximately 30% of patients undergoing radical prostatectomy, the presurgical prostate biopsy has been found to not be representative of the ground truth. Potential sources of this error were outlined earlier [8]. It is also worth highlighting that a degree of precision may be lost in studies that implemented systematic biopsy, rather than a targeted biopsy. In the former, the label applied to the segmented PCa may be inappropriately generalized from an adjacent cancer. The addition of an MRI-targeted biopsy is likely to increase the specificity of the biopsy for detecting clinically significant prostate cancer [176].

Given that the vast majority of the PCa radiomics literature is based on biopsy, the field has been predisposed to a significant bias. This could eventually be overcome with sufficient data, but the resource cost would be higher. The most likely resolution will be radiomic models validated on PCa biopsy results being externally validated on patient’s who proceeded to prostatectomy. If this is found not to be feasible, one could expect that prospective evaluations of imaging feature stability could exclusively feature prostatectomy patients. This approach will also have its limitations, as patient’s that proceed to prostatectomy may have different imaging features than those that are managed by active surveillance or radiotherapy-based management strategies.

A similar risk of bias exists at the level of PCa segmentation. Typically, radiomic analyses will only consider the molecular features (e.g., the GS and mutation status) of an index lesion, which is deemed the most aggressive site and likely origin of metastatic potential [177]. However, PCa is a multicentric disease. While recent work has suggested that this could allow for sampling of the whole gland to provide data to extract biomarkers [159], there is no data to suggest if the segmentation of all prostatic nodules or the whole gland could provide more (or less) reliable and relevant radiomic features. Segmentation of the whole gland could also lead to works that allow for the detection of more occult non-clinically significant PCa, allowing for non-invasive active surveillance approaches. Future prospective works would benefit from stating the intent to investigate such uncertainties a priori, allowing this potential source of observer bias to be challenged with greater methodological rigor.

Finally, there must also be a consideration for the limits of human understanding. The deep entropy radiomic features referred to earlier represent imaging characteristics without an intuitive function to a human user. Traditional rationalizations of covariate inclusion in a model imply that each covariate must have a role in the biological pathway to arrive at the clinical outcome. Based on the “black-box” nature of a CNN, there will not be an interpretable rationale why a selected imaging feature is associated with a clinical outcome [88], an ongoing initiative is now trying to address what is known as explainable AI (XAI) [178,179,180]. There will remain some residual uncertainty as to the viability of all such deep features, which may hinder later efforts of knowledge translation or medical device approvals.

Despite the prevailing influence of AI-based solutions (e.g., deep/machine learning) in the research community, the clinical use of such algorithms when it comes to predicting individual risk for any malignancy is still limited [181]. Increasing the detection of aggressive prostate cancer while decreasing unnecessary (false positive) biopsies was the rationale for an initiative that mildly penetrated the clinical settings in Scandinavian countries. The Stockholm 3 model (STHLM3) was derived from statistical inference algorithms and approximate-Bayesian computation, presenting an alternative to PSA testing.

With the adoption into routine clinical use in 2016, over 55,000 Swedish men were recruited to study the model’s efficacy [182,183]. It was then validated on nearly 10,000 men in Sweden, Norway, and Finland. Subsequent validation tests were carried out in Germany, the Netherlands, and the UK. Although, STHLM3 is a shallow example of machine learning, it is acceptable. In contrast, deep learning is still far from being widely adopted clinically but its potential yield is large. Instilling trust in deep learning approaches remains contingent on the reassurance of at least two issues, the establishment of the ethical AI/deep machine learning framework and the XAI accomplishment.

The dilemma that we face in most cases is that the best performing AI models are the least explainable due to the “black-box” decision of these models. Unlike in clinical settings, this has not presented a barrier to many commercial manufacturing outlets, where smart wearable devices have seen great consumer interest in the lay public. For risk prediction of chronic diseases, for instance, AI ethical standards and XAI are very important aspects to allow for the wide adoption of AI-based solutions. This raises another close term that is not to be confused with XAI-interpretability. Interpretability is the ability of an AI system to establish a cause–effect relationship. In contrast, the ability of this system’s inner parameters (i.e., deep learning usually exhibits millions of hidden parameters [88]) to explain its decision is its explainability [179].

## 6. Next Steps Involving AI with Radiomics

The specific radiomic applications for PCa will closely follow the clinical demands of the field, necessitating a multidisciplinary approach to understanding the underlying tissue characteristics, their relationship with -omics data, and the underlying computer science. Based on recently published work [86,88], incorporating additional clinical features can improve the quality of the model. Since the heterogeneity of PCa zones is dissimilar, it will likely be of value to observe the precedent set by PI-RADS and generate radiomic models specific to each zone [71]. As to clinical features, including relevant details to prostate cancer staging (e.g., T stage, PSA, and PSA doubling time) or tumor grade (e.g., finasteride use, ethnicity, and age) should be an ongoing consideration.

A pertinent example that did not benefit from the implementation of AI was the earlier referenced STHLM3 study, which benefitted from incorporating multiple clinical, biochemical, and radiological biomarkers [183]. Similarly, the radiogenomic models were seemingly improved with additional clinical data sources, but utilized conventional radiomic features in their modeling [47,157,165,182]. Enriching such models with deep radiomic features is a promising setting for study.

The greatest challenge is likely to be the generation of multicenter, large-sample, randomized-controlled clinical trials to validate a sufficiently stable model. Contributing to public datasets, such as The Cancer Genome Atlas (TCGA) [184], the Cancer Imaging Archive [185], and the Quantitative Imaging Network [186] will expedite earlier validation studies of radiomic models while improving generalizability. There is awareness of the considerable ethical concerns surrounding anonymization and informed consent of these Big Data investigations [187], with consensus guidelines prompting broad suggestions on how approaches to these issues should be documented [97,188].

A pragmatic next step would be refining the work to label the ROI through a deep learning approach (e.g., UNet, etc.) [127,189,190,191] and potentially implementing domain adaptation [192]. Moreover, there would be a greater amount of data available, as not every imaging data set would require biopsy data as well. Clinicians would be required to provide the ground-truth ROI in the labeling of the data sets. Deep learning models would be well suited to scale-up these large data sets to improve their performance.

The addition of new imaging modalities will expand the role of radiomics. The ability of PSMA-PET/CT to differentiate PCa from other soft tissues has already demonstrated superior performance in staging high-risk PCa, compared to CT and bone scan [193]. As these imaging techniques become more widely available, we will gain the ability to implement AI-based radiomic models that can incorporate data rich mpMRI, novel modalities, and hope for a collaborative big-data approach. The combination of all these directions will guide us closer to offering non-invasive personalized medicine.

## 7. Conclusions

This review highlighted the promising role of radiomics in predicting the GS in PCa. In a step-by-step fashion, the implementation of a radiomic pipeline was detailed alongside pertinent concerns. The fledgling role of AI-based approaches in predicting the GS was described, with suggested directions for future studies. By applying the potential power of these AI-based approaches to specific standardized prospective clinical studies, which can address concerns for imaging feature stability, the field of radiomics has the potential to undergo clinical translation in the near future. Until that time, the key challenge remains to ensure that the scientific community retains access to high-quality clinical and radiological resources so that the field has sufficient data to continue to mature.

## Figures and Tables

**Table 1:** Summary of Gleason score (GS) and International Society of Urological Pathology (ISUP) group.

Gleason Score	6 (3 + 3)	7 (3 + 4)	7 (4 + 3)	8 (4 + 4; 3 + 5; or 5 + 3)	9 (4 + 5; 5 + 4) or 10 (5 + 5)
ISUP Grade Group	1	2	3	4	5

**Table 2:** Summary of the area under the ROC curve (AUC) value for recently published papers related to GS prediction using radiomic signature derived from mpMRI of prostate cancer (PCa).

Reference	Feature Methods	GS ≤ 6	GS = 7	GS ≥ 7	GS ≥ 8	GS ≤ 7
Chaddad et al. [88]	Deep entropy features	88.82	87.45	82.28	93.03	84.72
Woznicki et al. [86]	^1^ Standard features + Shape + PI-RADS + PSAD + DRE	88.9	-	84.4	-	-
Li et al. [137]	^1^ Standard features + Clinical	-	-	98.00	-	-
Min et al. [138]	^1^ Standard features + Shape	82.30	-	-	-	-
Chaddad et al. [24]	^1^ Standard features	83.40	72.71	77.35	-	-
Cuocolo et al. [38]	Shape	78.00	-	-	-	-
Chaddad et al. [23]	Joint intensity matrices (JIM) + GLCM	78.40	82.35	64.76	-	-
Toivonen et al. [139]	GLCM + LBP + HOG + Gabor + Haar + filters	88.00	-	-	-	-
Jesen et al. [133]	^1^ Standard features	85.00	89.00	94.00	86.00	83.00
Cao et al. [140]	FocalNet	-	81.00	79.00	-	-

^1^ Standard features: Histogram + gray-level co-occurrence matrix (GLCM) + neighborhood gray-tone difference matrix (NGTDM) + Gray Level Size Zone Matrix (GLSZM), PSAD: prostate specific antigen density; DRE: digital rectal examination.

## Data Availability

No new data were created or analyzed in this study. Data sharing is not applicable to this article.
